# Development and Validation of a Clinical Practicum Assessment Tool for the NAACLS-Accredited Biomedical Science Program

**DOI:** 10.3390/ijerph19116651

**Published:** 2022-05-30

**Authors:** Taghreed Abunada, Atiyeh M. Abdallah, Rajvir Singh, Marawan Abu-Madi

**Affiliations:** 1 Department of Biomedical Science, College of Health Sciences, QU-Health, Qatar University, Doha P.O. Box 2713, Qatar; taghreed.abunada@qu.edu.qa (T.A.); aabdallah@qu.edu.qa (A.M.A.); 2 Cardiology Research, Heart Hospital, Hamad Medical Corporation, Doha P.O. Box 3050, Qatar; rsingh@hamad.qa

**Keywords:** medical laboratory sciences, students assessment, clinical practicum, tool validation, NAACLS

## Abstract

Student perspectives on their final year clinical placements in biomedical sciences at Qatar University are assessed using the clinical practicum assessment tool (CPAT), which was developed in-house following accreditation body requirements. The tool, which we call the CPAT-Qatar University (CPAT-QU), covers the three clinical practicum domains: practicum content, preceptors, and competencies. Here, we validate this tool. The CPAT-QU has 27 Likert-scale questions and free-text open questions. CPAT-QU readability was calculated using the Flesch–Kincaid Reading Ease (FKRE) instrument. Content validity was assessed using the average and universal average scale-level content validity indices (S-CVI/Average and S-CVI/UA). For construct validity, 50 employed graduates who had completed the practicum were consented for study participation, and the validity was calculated by a principal component analysis (PCA). Reliability was analyzed by Cronbach’s alpha. The S-CVI/Average and S-CVI/UA were 0.90 and 0.59, respectively, indicating that an adequate proportion of the content was relevant. The PCA extracted two core components, which explained 63% of the variance in the CPAT-QU. Cronbach’s alpha values for the items were within the acceptable range of 0.60–1.00, showing that internal consistency has a good level. CPAT-QU appears to be a useful tool for assessing student perspectives on their clinical placements; however, construct validity needs continuous improvement.

## 1. Introduction

The goal of clinical education in medical laboratory science (MLS) is to prepare graduates with professional competencies crucial for delivering clinical laboratory services. These competencies require the integration of knowledge, skills and attitudes to appropriately handle complex laboratory procedures and ensure that staff are effective contributors to rapidly changing healthcare systems [1,2,3]. Laboratory complexity is increasing due to constant and rapid scientific advances and technological innovations. These changes influence the educational requirements and qualifications needed for medical laboratory professionals [4], and high quality laboratory diagnostic services must be underpinned by a high standard of education [5]. Therefore, it is important to simultaneously enhance educational outcomes as well as clinical field training for MLS graduates and other allied health personnel. There is currently a worldwide shortage in the diagnostic workforce. For example, a recent study reported a shortfall of 840,000 diagnostic staff in the UK workforce [6]. Therefore, competency-based education is needed to ensure expansion of the diagnostic workforce with highly skilled and appropriately trained individuals.

The National Accrediting Agency for Clinical Laboratory Sciences (NAACLS), the American Society for Clinical Pathology (ASCP), and the American Society of Clinical Laboratory Sciences (ASCLS) proposed a set of standards to control the quality of MLS education and the profession [1,7]. Effective learning in the clinical setting depends on adequate supervision, mentoring, feedback, and assessment. Curriculum mapping and assessment matrices that align courses with desired program goals and outcomes are useful tools to monitor the quality of education delivered by MLS programs. Direct assessment methods, including exams, essays, presentations, classroom assignments, and capstone courses, can be used to asses students’ knowledge and skills, whereas indirect assessment methods, such as surveys and interviews, assess students’ reflections, opinions, and feelings about learning and the learning environment [8,9]. MLS programs commonly use three types of surveys: clinical experience surveys, graduate surveys, and employer surveys, which help to plan continuous quality improvement activities in collaboration with the main medical laboratory testing stakeholders.

The Department of Biomedical Sciences at Qatar University provides an NAACLS-accredited Biomedical Science bachelor’s degree program (http://www.qu.edu.qa/chs/biomedical-sciences (accessed on 1 January 2022). In the final year of the program, students are required to complete educational practicum courses at the two main hospitals in Qatar, Hamad Medical Corporation (HMC) and Sidra Medicine. To assess their clinical practicum experience, we developed an in-house tool, termed the Clinical Practicum Assessment Tool-Qatar University (CPAT-QU), based on NAACLS, ASCP, and ASCLS guidelines. The tool covers three aspects of training: the practicum content, the preceptors (trainers, instructors, and supervisors), and competencies. Despite it being common to use surveys to evaluate MLS clinical practicum training, we are not aware of a tool that has undergone comprehensive validation, even though fully validated tools would be more useful for continuous improvement and accreditation purposes. Hence, here, we evaluated the validity and reliability of the CPAT-QU survey tool used in the MLS-accredited program at Qatar University. Box 1 summarizes the key massages from this analysis.

Box 1 **Key Messages.**

**What is already known on this topic:** Tools are available to assess the quality of biomedical sciences practical courses in terms of content, preceptor quality, and competencies, because this information is essential for continuous quality improvement over time.
**What this study adds:** This clinical practicum assessment tool is the first tool to be validated for assessing student perspectives on their clinical placements in biomedical sciences.
**How this study might affect research, practice, or policy:** The clinical practicum assessment tool has value for continuous improvement and accreditation purposes and could help inform MLS program directors about ways to improve their clinical practicum based on high-quality evidence.

## 2. Materials and Methods

### 2.1. CPAT-QU Development and Description

**Figure 1:**
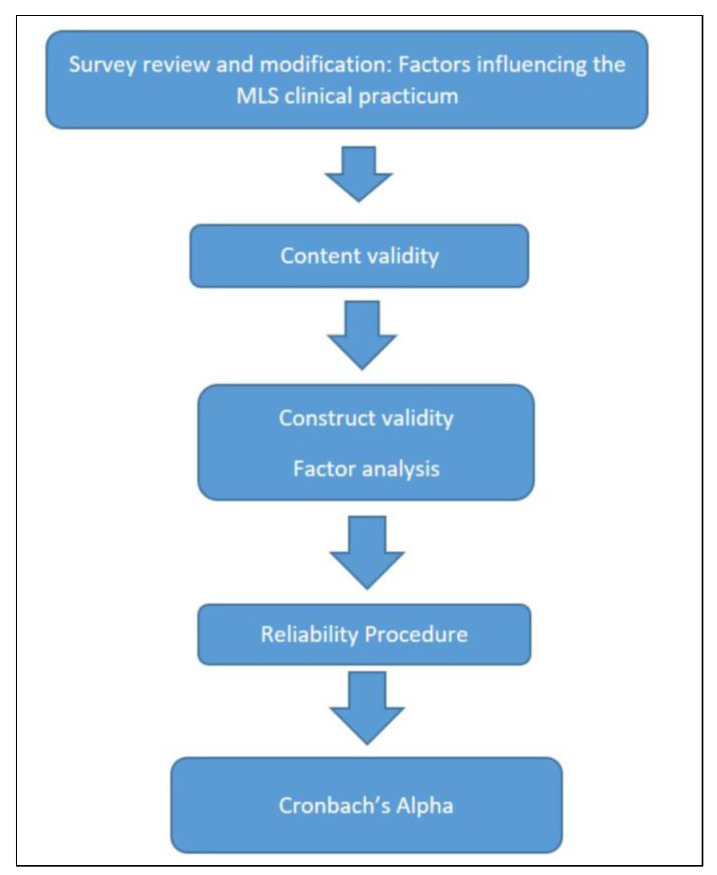
A flow chart depicting the process used for validating the CPAT-QU for the MLS clinical practicum.

The CPAT-QU was developed based on the NAACLS, ASCLS, and ASCP educational frameworks and guidelines [2,10,11,12]. In addition, we consulted training managers at the HMC and Sidra Medicine MLS clinical training sites. The tool has 27 Likert-scale questions as well as open-ended comment questions about students’ learning needs. It consists of three main domains (Table S1): practicum content, preceptors, and competencies. This tool is currently used by Department of Biomedical Sciences at QU to assess program graduate satisfaction with the clinical practicum. Entry-level competencies were categorized according to the three domains of learning (cognitive, psychomotor, and affective) by the research team and expert colleagues, as described by the ASCLS and ASCP (Table S2) [12,13]. Figure 1 shows a flow chart depicting the validation process.

A bipolar five-level Likert scale was used for domains 1 and 2: “very unsatisfied”, “unsatisfied”, “neutral/undecided”, “satisfied”, and “very satisfied”, coded into “−2”, “−1”, “0”, “1”, and “2”, respectively. A unipolar five-level Likert scale was used for competencies and skills required in workplaces (domain 3): “Not at all”, “little”, “to some extent”, “well”, and. “very well” coded into “0”, “1”, “2”, “3”, and “4”, respectively [14]. Qualitative responses were coded with integers for all items to make the responses equivalent to the quantitative responses for a unified statistical analysis. Domain scores were calculated by summing up the scores for all items in the domain. The individual index was calculated as:

Σ(item responses)/Σ(highest values in the items)

### 2.2. CPAT-QU Validation

#### 2.2.1. Readability

The Flesch–Kincaid Readability Ease (FKRE) and the Flesch–Kincaid Grade Level (FKRA) tests, the oldest and most reliable tests for readability reading in English [15], were used to calculate the understandability of the CPAT-QU. The FKRE formula is RE = 206.835 − (1.05 × ASL) − (84.6 × ASW), where RE = readability ease, ASL = average sentence length, and ASW = average number of syllables per word. The FKRA formula is (0.39 × ASL) + (11.8 × ASW) − 15.59, where FKRA = Flesch–Kincaid reading age, ASL = average sentence length, and ASW = average number of syllables per word.

#### 2.2.2. Content Validity

Six experts were selected based on their experience in clinical teaching, training, and MLS program accreditation standards to review the relevance of the CPAT-QU. All experts had at least 15 years of experience in the field. All experts had completed the College of American Pathology accreditation process for clinical sites in the State of Qatar. They had also participated in self-directed learning of the NAACLS accreditation. Three MLS experts were ASCP certified and actively maintained their certification. The experts were asked to comment on whether the tool’s content was appropriate for its purpose and for assessing the clinical practicum from the graduates’ perspective. The definitions of the three main domains and items were presented to the experts to rate each item independently using a 4-point ranking Likert scale: “Not relevant = 1,” “somewhat relevant = 2”, “quite relevant = 3”, and “highly relevant = 4” (Table S2). This scale was used to calculate the content validity index (CVI), scale-level content validity index using the average method (S-CVI/Average), and scale-level content validity using the universal average method (S-CVI/UA) indices based on the judgment of the six experts. These indices were computed, as they were easy to compute, understandable, and focused on the agreement of experts. Data analysis of content validity was performed using Microsoft Excel (Redmond, WA, USA).

#### 2.2.3. Construct Validity

A principal component analysis (PCA) was performed to calculate factor loading of the three domains (practicum content, preceptors, and competencies). A PCA is used to cluster items into different factors [16]. Eigenvalues greater than 1 were considered for factor loading. Cumulative percentages were used to explain the variance of the latent variable. A varimax rotation was used to maximize the sum of the variance by total rotation of the sum of squares, calculating the squared correlation between variables and factors. The total rotation of the sum of squared loadings was considered the variance after rotation attributable to each factor. A scree plot was used to determine how many factors to retain and flattening of the curve, which shows the eigenvalues on the x-axis and the number of factors on the y-axis. Kaiser–Meyer–Olkin (KMO) values were considered to measure sampling adequacy for the PCA.

#### 2.2.4. Reliability

Reliability was investigated by asking participants to answer the questionnaire twice with a 15–19-day gap between sessions. The same set of participants were given the questionnaire each time. The expectation was that there would be no substantial difference between the two outcomes [17].

### 2.3. Participants

The QU Institutional Review Board granted ethical approval (QU-IRB 1360-EA/20). The study targeted graduated students from the Biomedical Science program between 2015 to 2019 who are currently employed as Medical Laboratory Scientists/Technologists. A total of 150 students graduated and completed their clinical practicum during the specified period. The graduates were invited to participate by email and signed a consent form electronically via a Google link. Fifty (33%) employed graduates agreed to participate in this study. Initially, we were planning face-to-face interviews with the students, however; due to the COVID-19 pandemic, telephone interviews were performed. After obtaining informed consent, two telephonic interviews were conducted in English no more than 19 days apart by a trained researcher. Interviews were not audio recorded. Data were collected over a two-month period (September–October 2020), and the outcomes were entered into an Excel spreadsheet.

### 2.4. Statistical Analysis

A PCA was used for factor analysis using the principal factor method with a varimax rotation to test the hypothesized domain structure. KMO values ≥ 0.8 were used to establish an appropriate sample size for the analysis. The Kaiser criterion was used to select factors with eigenvalues ≥ 1, and scree plot/graphs were used to illustrate the descending variances for factor extraction. A Cronbach’s α coefficient of ≥0.7 was considered internally consistent. A two-tailed *p*-value of ≤0.05 was considered significant. IBM SPSS 26.0 statistical package (IBM Statistics, Armonk, NY, USA) was used for the analysis.

## 3. Results

### 3.1. Readability and Understandability

To determine whether the questionnaire was understandable by people of different educational levels, readability statistics were calculated. The FKRE score for the questionnaire was 30.9, which indicates that the survey could easily be understood by college graduates. The FKRA was 11.6, suggesting that the survey would be understandable to anyone with a grade-10-level education.

### 3.2. Content Validity

The survey’s content validity was assessed based on expert evaluations of item relevance. A few items in the survey were modified according to the experts’ suggestions to create the final version used in the study. CVI, S-CVI/Average, and SCVI/UA were chosen for their advantages in terms of computation, understandability, and agreement on relevance. The comprehensiveness and representativeness of content were measured on a scale to determine its validity. The S-CVI/Average was 0.90 and S-CVI/UA was 0.59, whereas the total agreement was 16 for 27 items, indicating adequate survey validity (Table 1).

### 3.3. Construct Validity

**Figure 2:**
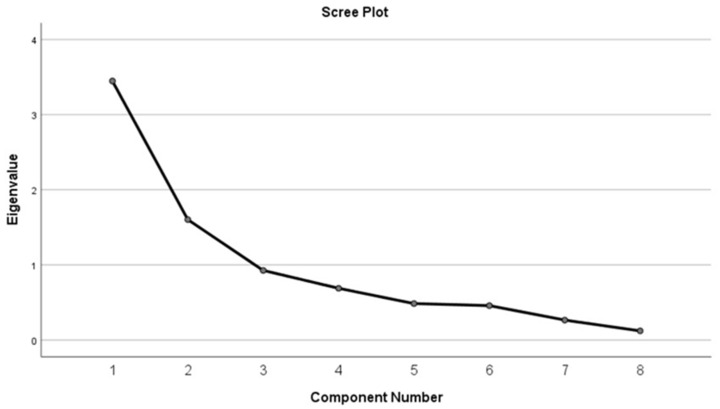
Scree plot for the principal component analysis (PCA) for the CPAT-QU construct validity. The descending tendency became weak from the third point.

PCA of the 27 questionnaire items showed that 63% of the variation in the latent variable was explained by two components (Table 2). All variants in Table 3 were in the first component, whereas the second component only contained variant numbers 4, 6, and 8. Results were further illustrated by the scree plot. Visual inspection of the scree plot shows that the point of inflexion in the plot occurred at the third factor, indicating that two factors should be retained (Figure 2).

### 3.4. Reliability

Reliability refers to ensuring that the various items measuring the different constructs of a test deliver consistent results. Consistency in the items was measured by Cronbach’s α. The values were 0.80, 0.76, 0.89, 0.81, 0.85, 0.86, and 0.92, respectively, falling within the acceptable range of 0.60–1.00 and indicating good internal consistency between the different variables (Table 4).

## 4. Discussion

The purpose of this study was to validate the CPAT-QU tool used for MLS clinical practicum assessment from the graduate perspective. The CPAT-QU was developed based on the NAACLS, ASCLS, and ASCP educational framework and the three categories of MLS entry-level competencies expected to prepare graduates for MLS practice. Our results indicate that the CPAT-QU has an acceptable content validity and relevance, as the S-CVI/Average was 0.93. The tool captures 63% of the variance and explains two core components with eigenvalues greater than one. It has a good level of internal consistency and reliability, as Cronbach’s α scores fell within the acceptable range of 0.60–1.00.

The expert analysis showed that preceptors play an important role in the practicum experience. Preceptor attitude, their ability to convey knowledge, and their interest in clinical teaching were significant factors contributing to the overall satisfaction of MLS graduates toward the clinical practicum. Supervisory level, feedback, and formal competency assessments may be extremely variable in different student’s clinical experiences, as reported by others [18,19]. Preceptors expert in teaching, training, and adult learning styles are essential to maintain the overall quality of the clinical practicum. Having sufficient, efficient, and well-qualified MLS preceptors is often a major challenge for MLS program directors [20,21]. In a systemic review analysis, Griffiths et.al. found that the structure and content intervention of preceptors lacked rigor in outcome measurement [22]. This highlights the importance of measuring the impact of educational interventions on broader outcomes, such as quality of client care. It is particularly essential to promote professional socialization and to develop the professional identity of the newly graduated MLS students during their transition from the classroom into the medical laboratory workforce [23]. Other studies have shown that clinical rotation sites, work environment, and employment benefits influence MLS graduates’ satisfaction levels and attitudes toward their MLS clinical training, recruitment, and even future job selection [24,25,26].

We took every precaution to construct the CPAT-QU tool based on the cognitive, psychomotor, and affective domains addressed by the NAACLS, ASCLS, and ASCP, and it was able to capture 63% of the variance with two core components. Indeed, many committees and task forces from the NAACLS, ASCLS, and ASCP have provided road maps to help MLS programs develop well-constructed assessment plans for entry-level competencies and their effective contribution toward graduate employability. It is also well recognized that the quality of clinical education and training reflected in such assessment plans should be based on surveys [4,12,13]. Such surveys, which are widely used by MLS programs, might serve the goal of complying with accreditation bodies, but they might need revision to ensure their continuing validity. Therefore, we recommend investigating this tool in other institutional settings to further study its validity. To our knowledge, this is the first study to assess tool validity for the MLS clinical practicum, a major core of these educational programs. We believe that this study has significant implications for MLS program leaders and could help to inform MLS program directors about ways to improve their clinical practicum. Our data suggest that unperceived aspects influence the quality of the MLS clinical practicum. Exchange of knowledge, experience, and tools within the MLS program community would be beneficial in this regard. Information and knowledge about the procedures of the practicum and detailed explanations about activities are also important for students. A survey among nursing graduates found that providing concrete guidance about the training helped them to develop the required skills [27].

Our study has some limitations. First, all the participants were selected from a single educational institution, Qatar University, which may have resulted in selection bias. However, there is no other NAACLS-accredited program in Qatar. Second, no “gold standard” tool for assessing the clinical practicum is available. Therefore, we could not compare our results with other tools or perform cross-cultural validation, and the results may not be generalizable. Third, this assessment covered only the student perspective and not preceptor/trainer experience, knowledge, and attitudes. Lastly, the KMO measure of sampling adequacy was 0.61, so more subjects are need for questionnaire validation, and a confirmatory factor analysis should be conducted to confirm that the items accurately reflect the underlying constructs.

## 5. Conclusions

The CPAT-QU is a reliable tool that captures 63% of the variance in the MLS clinical practicum from the graduates’ perspective. Future work will be focused on increasing the construct validity of the survey. Moreover, to further improve tool validity and reliability, we recommend investigating this tool in other institutional settings to improve the reliability of the survey.

## Figures and Tables

**Table 1:** CPAT-QU items with inadequate universal agreement among the experts.

Domain	Item *	Experts in Agreement (N)	Relevance CVI
Content of MLS clinical practicum	1. Organization	6	1.00
	2. Content of the clinical training rotations	6	1.00
	3. Evaluation criteria of the clinical training rotations	6	1.00
	4. Length of the clinical training rotations	6	1.00
Preceptors mastering MLS clinical practicum	1. Instructor’s attitude	5	0.83
	2. Command of material, knowledge, and expertise	6	1.00
	3. Ability to convey knowledge and expertise	5	0.83
	4. Interest in clinical teaching and training	5	0.83
Skills and competencies of MLS clinical practicum	1. Recall of basic knowledge and comprehension	6	1.00
	2. Awareness of organizational structure, lab management, safety, infection prevention control measures, and quality	6	1.00
	3. Awareness of financial management, budget, lab staffing, HR laws, and regulation of the degree profession	3	0.50
	4. Application and interpretation of content or lab result	6	1.00
	5. Critical analysis, decision-making, problem solving	5	0.83
	6. Ability to retrieve/locate information from a range of sources	6	1.00
	7. Readiness; an awareness of and ready to analyze samples or observe	6	1.00
	8. Competence and confidence with performing a task or analyzing samples	6	1.00
	9. Proficiency and adaptation, ability to alter performance successfully	6	1.00
	10. Research skills, such as planning and designing experiments	4	0.67
	11. Using technology in communication skills and information exchange	5	0.83
	12. Report writing and written communication skills	6	1.00
	13. Oral presentation and verbal communication skills	4	0.67
	14. Appreciation of ethical scientific behavior	5	0.83
	15. Leadership skills	4	0.67
	16. Team working skills	6	1.00
	17. Time management and organizational skills	6	1.00
	18. Ability to have and use own initiative	6	1.00
	19. Possession of independent learning required for continuing professional development	5	0.83
	S-CVI/Average		0.90
	Total agreement		16.00
		S-CVI/UA	0.59

* Numbers indicate the item order as listed in the questionnaire.

**Table 2:** Factor analysis for construct validity.

Component	Initial Eigenvalues			Rotation Sums of Squared Loadings
	Total	Variance %	Cumulative %	Total
1	3.448	43.102	43.102	2.852
2	1.602	20.029	63.131	2.198

**Table 3:** Principal component analysis of the questionnaire variables matrix. All variables were positive in the first component, and only 4, 6 and 8 were positive in the second component.

Variables	Component	
	1	2
1. Content of the clinical practicum	0.609	−0.076
2. Instructors mastering the clinical practicum	0.565	−0.252
3. Cognitive knowledge and skills developed during the clinical practicum	0.773	−0.416
4. Cognitive knowledge and skills used in the degree profession	0.566	0.589
5. Psychomotor skills developed during the clinical practicum	0.826	−0.309
6. Psychomotor skills used in the degree profession	0.623	0.649
7. Affective skills developed during the clinical practicum	0.671	−0.429
8. Affective skills used in the degree profession	0.566	0.558

**Table 4:** Internal consistency of the questionnaire domain indices.

Variable	Cronbach’s Alpha
Content of the clinical practicum	0.80
Instructors mastering the clinical practicum	0.76
Cognitive knowledge and skills developed during the clinical practicum	0.89
Cognitive knowledge and skills used in the degree profession	0.76
Psychomotor skills developed during the clinical practicum	0.81
Psychomotor skills used in the degree profession	0.85
Affective skills developed during the clinical practicum	0.86
Affective skills used in the degree profession	0.92

## Data Availability

Not applicable.

## Supplementary Materials

The following supporting information can be downloaded at: https://www.mdpi.com/article/10.3390/ijerph19116651/s1. Table S1: CPAT-QU Questionnaire; Table S2: Description of CPAT-QU tool Domains/constructs.
