# Supplementary material for: Development and Validation of a Clinical Practicum Assessment Tool for the NAACLS-Accredited Biomedical Science Program
Source: Int J Environ Res Public Health. 2022 May 30;19(11):6651. doi: 10.3390/ijerph19116651 (PMC9180805; doi:10.3390/ijerph19116651)
Supplement: Supplementary file 1 [file ijerph-19-06651-s001.zip › ijerph-1701642-supplementary.pdf]

Table S1 CPAT-QU Questionnaire

**Demographic**	
Graduation	Year ______________ Semester ______________
Educational level	
Profession status	☐ Employed ☐ engaged in training
If employed, Job title	
Joining date	
Employer name	
Lab Specialty or section(s)	☐ Hematology ☐ chemistry ☐ immunology<br>☐ Virology ☐ Microbiology ☐ Blood Bank<br>☐ Molecular/Genetics ☐ Research lab
How satisfied are you with the **CONTENT** of the Clinical Training Internship Program offered by Qatar University in term of?<br>**"0 = very unsatisfied, 1 = unsatisfied, 2 = neutral/undecided, 3= satisfied, 4 = very satisfied"**	
**Organization**	
1. Objectives were clearly stated. Written procedures were readily available.	
**Content**	
2. Material was presented at an appropriate difficulty level. Instruction enabled me to understand & utilize procedures. There were sufficient samples available to perform, repeat and practice the required procedures.	
**Evaluation criteria**	
3. The grading system was clearly explained. Exams and assignments were fair.Exams reflected the objectives & rotations' content.	
**Length**	
4. The amount of time allocated for the rotations was appropriate. Time was well utilized.	
How satisfied are you with the **CLINICAL INSTRUCTORS** of the Clinical Training Internship Program offered by Qatar University in term of?<br>**"0 = very unsatisfied, 1 = unsatisfied, 2 = neutral/undecided, 3= satisfied, 4 = very satisfied"**	
**Instructor's Attitude**	
5. Instructor made you feel welcome and part of the laboratory team. Instructor exhibited openness to student ideas and questions. Instructor inspired me to put forth my best effort and had a positive attitude.	
**Command of Material/Knowledge/Expertise**	
6. Instructor appeared knowledgeable in the subject matter.	
**Ability to Convey Knowledge and Expertise**	
7. Instructor communicated the subject matter well. Instructor perceived student's ability and adjusted pace accordingly. Instructor presented information in an interesting and thought-provoking manner.	
**Interest in Teaching**	
8. Instructor appeared to be interested in teaching students. Instructor demonstrated enthusiasm	

With respect to your undergraduate degree, please indicate how well the clinical training internship assisted you in **DEVELOPING** the knowledge and skills required in your profession and how well such knowledge and skills were **USED** in your current profession?<br>**"0 = Not at all, 1 = little, 2 = to some extent, 3 = well, 4 = very well"**		
**Knowledge/skills**	**Internship Efficiency score in term of developed knowledge/skills During undergraduate**	**Internship Efficiency score in term of used knowledge/skills in your degree profession**
**Cognitive Domain**		
9. Recall of basic knowledge and comprehension		
10. Awareness of organizational structure, management, safety, infection prevention control measures, quality management		
11. Awareness of financial management, budget, staffing, HR laws and regulation of the degree profession.		
12. Application and interpretation of content/results		
13. Critical analysis, decision-making, and problem solving, which relates to the evaluation and processing of knowledge.		
14. Ability to retrieve/locate information from a range of sources		
**Psychomotor Domain**		
15. Readiness; an awareness of and ready to analyze samples or observe		
16. Competence and confidence with performing a task or analyzing samples		
17. Proficiency and adaptation, ability to alter performance successfully when encountering unexpected lab results or having new situations		
18. Research skills (e.g. planning and design experiments)		
19. Information and communication technology skills		
20. Report writing and written communication skills		
21. Oral presentation and verbal communication		
**Affective Domain**		
22. Appreciation of ethical scientific behavior		
23. Leadership skills		
24. Team working skills		
25. Time management and organizational skills		
26. Ability to use own initiative		
27. Ability of independent learning required for continuing professional development		
How may the program improve its **CURRICULUM** to match the need of the labor market more from your point of view (open question)?		

Table S2 Description of CPAT-QU tool Domains/constructs

Domain 1 : Satisfaction level of the graduate with the content area of the clinical practicum	Number of Items	Description	
Organization of CP program	1	The CP program objectives were clearly stated, written procedures were readily available.	Satisfaction with (coded: Very satisfied =+2 to Very unsatisfied = - 2)
Content of CP program	1	The material was presented at an appropriate difficulty level, instruction enabled you to understand & utilize procedures, and there were sufficient samples available to observe, perform, repeat and practice the required procedures.	Satisfaction with (coded: Very satisfied =+2 to Very unsatisfied = - 2)
Evaluation criteria of CP program	1	The grading system was clearly explained. Exams and assignments were fair exams reflected the objectives & rotations' content.	Satisfaction with (coded: Very satisfied =+2 to Very unsatisfied = - 2)
Length of CP program	1	The amount of time allocated for the rotations was appropriate. Time was well utilized.	Satisfaction with (coded: Very satisfied =+2 to Very unsatisfied = - 2)

Domain 2: Satisfaction level of the graduate with the Preceptors Mastering the clinical Practicum	Number of Items	Description	
Instructor's attitude and environment	1	Instructor made you feel welcome and part of the laboratory team. Instructor exhibited openness to student ideas and questions and had a positive attitude. Instructor inspired me to put forth my best effort.	Satisfaction with (coded: Very satisfied =+2 to Very unsatisfied = - 2)
Command of Material/Knowledge/Expertise	1	Instructor appeared knowledgeable in the subject matter.	Satisfaction with (coded: Very satisfied =+2 to Very unsatisfied = - 2)
Ability to Convey Knowledge and Expertise	1	Instructor communicated the subject matter well. Instructor perceived student's ability and adjusted pace accordingly. Instructor presented information in an interesting and thought-provoking manner.	Satisfaction with (coded: Very satisfied =+2 to Very unsatisfied = - 2)
Interest in Teaching & Training	1	Instructor appeared to be interested in teaching students. Instructor demonstrated enthusiasm	Satisfaction with (coded: Very satisfied =+2 to Very unsatisfied = - 2)

Domain 3: Wellness level of entry level competencies development at undergraduate study time and their utilization in the degree profession	Number of Items	Description	
Cognitive Domain	6	1. Recall of basic knowledge and comprehension.<br>2. Awareness of organizational structure, management, safety, infection prevention control measures, quality management.	Wellness of knowledge and skills development and utilization (coded: Not at all = 0, little = 1, to some extent = 2, well = 3, very well =4)

		3. Awareness of financial management, budget, staffing, HR laws and regulation of the degree profession.<br>4. Application and interpretation of content/results<br>5. Critical analysis, decision-making, and problem solving, which relates to the evaluation and processing of knowledge.<br>6. Ability to retrieve/locate information from a range of sources	
Psychomotor Domain	7	1. Readiness; an awareness of and ready to analyze samples or observe.<br>2. Competence and confidence with performing a task or analyzing samples.<br>3. Proficiency and adaptation, ability to alter performance successfully when encountering unexpected lab results or having new situations.<br>4. Research skills (e.g. planning and design experiments).<br>5. Information and communication technology skills.<br>6. Report writing and written communication skills.<br>7. Oral presentation and verbal communication.	Wellness of knowledge and skills development and utilization (coded: Not at all = 0, little = 1, to some extent = 2, well = 3, very well =4)
Affective Domain	6	1. Appreciation of ethical scientific behavior.<br>2. Leadership skills.<br>3. Team working skills.<br>4. Time management and organizational skills.<br>5. Ability to use own initiative.<br>6. Ability of independent learning required for continuing professional development	Wellness of knowledge and skills development and utilization (coded: Not at all = 0, little = 1, to some extent = 2, well = 3, very well =4)
